# Incidence of cognitive impairment after hypothetical interventions on depression, nighttime sleep duration, and leisure activity engagement among older Chinese adults: An application of the parametric g-formula

**DOI:** 10.3389/fpubh.2023.1088833

**Published:** 2023-02-15

**Authors:** Nan Zhang, Fenghui Chen, Cui Wang, Ping Yan

**Affiliations:** ^1^Department of Surgical Nursing, School of Nursing, Xinjiang Medical University, Urumqi, Xinjiang, China; ^2^Department of Internal Medicine Nursing, School of Nursing, Xinjiang Medical University, Urumqi, Xinjiang, China; ^3^Department of Health Science, School of Nursing, Peking University, Beijing, China

**Keywords:** older adults, cognitive impairment, sleep duration, depression, leisure activity, g-formula

## Abstract

**Background:**

Cognitive impairment is an age-relevant intermediate stage where cognition declines to a state between the normal aging process and dementia. Earlier studies reported that depression, inappropriate nighttime sleep duration (NSD), and limited leisure activity engagement are cognitive impairment risk factors among older adults. Thus, we postulated that interventions on depression, sleep duration, and leisure activity engagement can reduce cognitive impairment risk. However, no earlier research ever explored this.

**Methods:**

The data of 4,819 respondents aged 60 years and above, without cognitive impairment at baseline and with no prior history of memory-related diseases, including Alzheimer's disease, Parkinson's disease, and encephalatrophy, were obtained from the China Health and Retirement Longitudinal Study (CHARLS) between 2011 and 2018. The parametric g-formula, an analytic tool for estimating standardized outcome distributions using covariate (exposure and confounders)-specific estimates of the outcome distribution, was used to estimate 7-year cumulative cognitive impairment risks among older Chinese adults, under independent hypothetical interventions on depression, NSD, and leisure activity engagement, which was subdivided into social activity (SA) and intellectual activity (IA) for the different intervention combinations.

**Results:**

The observed cognitive impairment risk was 37.52%. Independent intervention on IA was the most effective factor in reducing incident cognitive impairment, with a risk ratio (RR) of 0.75 (95% confidence interval [CI]: 0.67–0.82), followed by depression (RR: 0.89, 95% CI: 0.85–0.93) and NSD (RR: 0.88, 95% CI: 0.80–0.95). The joint intervention combining depression, NSD, and IA interventions could reduce the risk by 17.11%, with an RR of 0.56 (95% CI: 0.48–0.65). In subgroup analyses, independent interventions on depression and IA had analogously significant effects on men and women. However, interventions on depression and IA had stronger effects on literate than illiterate individuals.

**Conclusions:**

Hypothetical interventions on depression, NSD, and IA reduced cognitive impairment risks among older Chinese adults, both independently and jointly. The findings of the present study suggest that the intervention measures on depression, inappropriate NSD, limited intellectual activities, and their combination may prove to be effective strategies for preventing cognitive impairment among older adults.

## Introduction

Cognitive impairment is an age-relevant intermediate stage of cognitive decline between the normal aging process and dementia, featuring cognition declines in memory, visuospatial ability, orientation, calculation, execution, and comprehension (1, 2). Older adults with cognitive impairment tend to have a significantly higher risk of dementia, with a progression rate of 10%−30% per year, whereas those adults without cognitive impairment have a progression rate of 1%−2% annually (3). Accordingly, it is important to identify older adults with cognitive impairment not only to develop interventions that alleviate individual suffering but also because this represents a population that is at an increased risk of developing dementia. Among older adults aged 60 years and above, the global prevalence of cognitive impairment ranges from 5.1% to 41.0% (4). In China, it is reported that the prevalence of cognitive impairment varies from 2.40% to 39.88% among older adults in different provinces, and approximately 36,000 additional cases of cognitive impairment occur annually (2, 5). The Chinese population is aging dramatically. In 2021, the population of people aged 60 years and above was 267 million, but it is projected to surpass the 400 million mark by 2035 (6). Therefore, China faces several challenges with regard to cognitive impairment among older adults, and early screening and intervention for cognitive impairment should receive more attention.

Evidence shows that women and individuals with low education levels are common risk factors for cognitive impairment (7, 8). An earlier study showed that overall cognitive impairment is more serious in women than in men (9). Consistent with this result, another study confirmed that Chinese women are significantly disadvantaged when it comes to cognitive functioning in old age (10). As regards education, a Chinese-based longitudinal study revealed that a low education level is associated with a high risk of cognitive impairment among older adults (5). As well as gender and education level, depression, inappropriate nighttime sleep duration (NSD), and limited leisure activity engagement are reported as other risk factors for cognitive impairment among older adults (11–13). Obvious issues with addressing the immutable characteristic of gender, and difficulty with changing the education level, especially in older adults, are prevalent. However, numerous studies revealed that treatments or interventions on depression, inappropriate NSD, and limited leisure activity engagement can not only ameliorate the symptoms themselves but may also be beneficial for improving cognitive performance and reducing the risk of cognitive impairment, although the results of these studies are mixed. For depression, reviews by Motter et al. (14) and Culpepper et al. (15), which examined the effectiveness of cognitive remediation therapy (CRT) in improving depression and cognition, revealed that CRT is effective in reducing depressive symptoms and improving cognitive functions. However, Wong et al. (16) and Adler et al. (17) demonstrated that, in cognitively impaired patients with depression, treatments are only effective in improving depression and not cognitive impairment. As regards the NSD, earlier studies reported that changes from long (≥9 h) to moderate (6–9 h) sleep duration (18), or from short (< 6 h) to moderate (6–9 h) sleep duration (19), were negatively associated with cognitive impairment risks. Nevertheless, three other studies demonstrated that, compared with unchanged sleep durations in baseline and follow-ups, any increases or decreases in sleep durations were associated with worse cognitive performances or increased cognitive impairment risks, despite using the different sleep duration references (20–22). Participation in leisure activity and its subtypes, namely social activity (SA), physical activity (PA), and intellectual activity (IA), has been proven to be beneficial for the preservation of cognitive function (23). Several studies examining the association between leisure activity engagement and cognitive function in older adults demonstrated that SA and/or IA are relevant to decreased cognitive deficit and risks of dementia (24–26). Notably, changes in leisure activity engagement also have effects on subsequent trajectories of age-related cognitive performance, so individuals with more leisure activity engagement than before will have slower cognitive decline rates as they age (13). Although some, mostly observational, studies confirmed the risk factors of cognitive impairment to a certain extent, many limitations are prevalent with regard to not properly controlling confounding variates such as time-varying confounders in observational studies and a lack of, or inability to, estimate the causal effect of risk factors.

The standard for evaluating the comparative effectiveness and the causal effect of an intervention is a randomized controlled trial (RCT). However, when RCTs are not feasible or timely, the impact of potential treatment strategies can be informed empirically using observational data to emulate a target trial (27). Target trial emulation is the application of design principles from RCTs to the analysis of observational data, thereby explicitly tying the analysis to the trial it is emulating (28). This requires a clear specification of the trial protocol elements and, when assessing interventions sustained over time, an analytic method known as the g-formula is required to appropriately account for time-dependent confounding variate (27). The g-formula, first described by Robins (29) in 1986, is used to estimate the causal effect of arsenic on heart disease in an occupationally exposed cohort and is an analytic tool for estimating standardized outcome distributions using covariate (exposure and confounders)-specific estimates of the outcome distribution (30). It can be viewed as a generalized form of standardization of the conditional hazard under each treatment strategy to the joint distribution of the time-varying covariates so that it can appropriately adjust for time-varying confounders affected by prior exposures. It is especially well-suited to estimating effects when the intervention involves multiple factors (joint interventions) or decisions that depend on the value of evolving time-dependent factors (31). Moreover, while observational analyses are restricted to a framework where one can only test interventions that have been explicitly carried out in the data, the g-formula enables one to simulate treatment strategies (also called hypothetical intervention) and to estimate their effect, even if those strategies have not been fully carried out in the data used to construct the model (32). Earlier studies using the parametric g-formula were conducted to measure hypothetical interventions on the levels of blood pressure, cholesterol, weight, PA, smoking, and alcohol intake for preventing stroke (33) and to estimate the risk of fall under single and joint interventions on sleep duration, SA, smoking, drinking, body mass index (BMI), systolic blood pressure, vision, depression, and activities of daily living scores (ADLs) (34). However, research using the parametric g-formula to investigate the risks of cognitive impairment under hypothetical interventions is limited. Therefore, the present study aimed at estimating the 7-year cumulative risks of cognitive impairment under independent interventions on depression, NSD, SA, and IA, as well as joint interventions consisting of different combinations thereof, using the parametric g-formula on the basis of a nationwide representative cohort of the China Health and Retirement Longitudinal Study (CHARLS). Such an estimation can help guide and provide intervention strategies and alternative treatments for the prevention of cognitive impairment among community-dwelling older Chinese adults.

## Materials and methods

### Data sources and participants

Data were extracted from four waves of CHARLS conducted in 2011 (wave 1), 2013 (wave 2), 2015 (wave 3), and 2018 (wave 4). CHARLS is a national longitudinal survey aimed at investigating and evaluating the aging issue of adults from 150 country-level units distributed in 28 provinces of China. It has been conducted by the Institute of Social Science Survey of Peking University since 2011, which updates the aforementioned survey every 2 years. The participants were informed of the research purpose and signed written informed consent forms to participate. Ethical approval was obtained from the Biomedical Ethics Review Committee of Peking University (IRB00001052-11015) (35).

**Figure 1:**
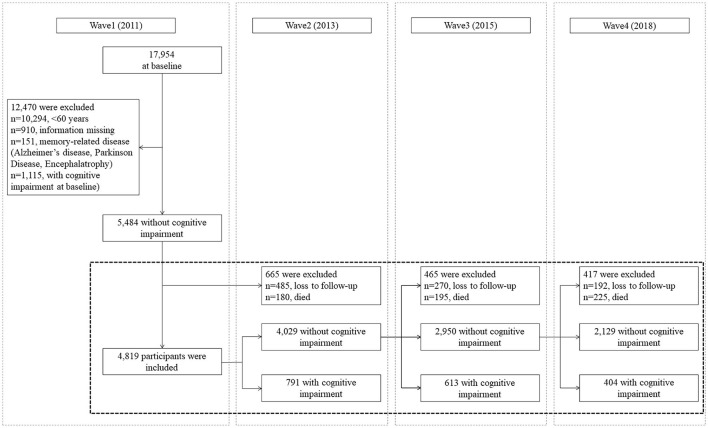
Sample selection flowchart based on the 2011–2018 China Health and Retirement Longitudinal Study (CHARLS) data.

In total, 17,954 respondents were included in the first wave (2011), and these data were used as the baseline. To emulate hypothetical interventions on cognitive impairment risks, the inclusion and exclusion criteria were set according to the g-formula principle. The inclusion criteria of the present study were as follows: (1) participants aged 60 years and above, (2) participants who had no cognitive impairment at baseline, and (3) participants who had completed at least one follow-up. The exclusion criteria of the present study were as follows: (1) participants' key variables were missing and (2) participants were diagnosed with memory-related diseases including Alzheimer's disease, Parkinson's disease, and encephalatrophy at baseline. Finally, a total of 4,819 respondents were included in the analysis. The sample selection flowchart for this study is presented in Figure 1. For each participant, the follow-up was ended at the time of onset of cognitive impairment, when the patient was lost to follow-up, or when the patient was part of the examination of wave 4 (2018), whichever occurred first.

### Cognitive impairment

The cognitive function was assessed by four dimensions comprising orientation, attention, episodic memory, and visuospatial ability. Cognition scores were calculated using the Telephone Interview for Cognitive Status (TICS-10), word recall, and picture redrawing. An overall cognition score (36) was considered the primary outcome of interest. The sum of all three TICS-10 scores (orientation and attention), word recall (episodic memory), and figure drawing (visual spatial ability) was then calculated to assess the global cognitive function, with the score ranging from 0 to 21. A higher score indicated a better cognitive function. Cronbach's α was 0.84 across all four waves.

Due to the discordancy of the criteria among multiple measurements, we used the concept of aging-associated cognitive decline (AACD) to define cognitive impairment, requiring at least one standard deviation (SD) below age-appropriate norms (10, 37). AACD—which is recommended by the International Psychogeriatric Association in collaboration with the World Health Organization (the WHO)—seeks evidence of cognitive decline within a broader range of cognitive domains, covering all criteria for estimating cognitive impairment. The AACD has been widely used as diagnostic criteria for estimating cognitive decline by both clinicians and scholars, therefore fitting the aim of this study (38). Respondents were divided into age groups, with each group representing a 5-year interval, and individuals with cognition scores lower than 1 SD from their group mean were defined as having cognitive impairment.

### Intervention variables

Depressive symptoms were assessed using the 10-item Center for Epidemiologic Studies Depression (CES-D-10) Scale which comprised 10 depressive effects: bothered by things that usually did not bother them, trouble in concentration, feeling depressed, effort exerted to complete daily tasks, feeling fearful, restless sleep, loneliness, inability to “get going,” feeling hopeful about the future, and feeling happy. Each of the 4-option responses to the item was scored from 0 to 3, and the total score was the sum of points for all 10 items. A total score of 10 or higher indicated clinical depression (39).

Nighttime sleep duration was obtained from the question “During the past month, how many hours of actual sleep did you get at night?” As reported in earlier studies, we took an NSD of 6 h as the reference duration (12) and classified participants into a 6-hr group and a non-6-hr group.

Leisure activity engagement was assessed through SA and IA (24). The SA items included: interacting with friends; going to a sport, social, or any other kind of club; taking part in a community-related organization; and doing volunteer or charity work. The IA items included: playing Mahjong, chess, or cards; attending an education or training course; stock investment; and surfing the Internet. The frequency of each activity was rated as never (score = 0), not regularly (score = 1), almost every week (score = 2), and almost daily (score = 3). All activity types were synthesized to a sum score ranging from 0 to 12 based on the frequency level. Participants whose sum score was > 1 were categorized as “participating in SA/IA”; otherwise, they were regarded as “not participating in SA/IA.”

### Covariates

Baseline covariates included gender (male/female), age (continuous), education level (literate/illiterate), and marital status (married/other). Time-varying covariates included ADLs (restricted/unrestricted), instrumental activities of daily living scores (IADLs, restricted/unrestricted), chronic diseases (0/1–2 diseases/ ≥ 3 diseases), smoking status (none/quit/still smokes), drinking status (none of these/drinks but less than once a month/drinks more than once a month), and PA (continuous).

Functional disability was measured based on ADLs and IADLs. ADLs included six aspects: dressing, bathing or showering, eating, getting into or out of bed, using the toilet, and controlling urination and defecation. IADLs comprised five aspects: doing household chores, preparing hot meals, shopping, taking medications, and managing money. The response scale contained four options: “no, I do not have any difficulty,” “I have difficulty but can still do it,” “yes, I have difficulty and need help,” and “I cannot do it.” Participants whose responses were “no, I do not have any difficulty” for all items of ADLs or IADLs were classified as “unrestricted” to ADLs or IADLs; otherwise, they were classified as “restricted.” Chronic diseases were determined by asking “Have you been diagnosed with the following conditions by a doctor?” and comprised the following 13 diseases: hypertension, dyslipidemia, diabetes, or hyperglycemia; cancer or malignant tumor; chronic lung disease; liver disease; heart attack; coronary heart disease; angina; congestive heart failure; stroke; kidney disease; stomach, emotional, nervous or psychiatric problems; arthritis or rheumatism; and asthma. Participants were classified as having no diseases, one to two chronic diseases, and over two chronic diseases based on their responses. PA comprised the amount of time a person spent on vigorous activities, moderate activities, and walking in a usual week. According to the responses, we indexed the amount of PA per day as 1 (< 0.5 h), 2 (0.5–2 h), 3 (2–4 h), and 4 (>4 h). The weekly PA duration score was calculated by multiplying the number of days the activity was performed and the daily PA duration index for each activity. Finally, we generated the PA score variables using metabolic equivalent (MET) multipliers as follows: PA score = 8.0 × total vigorous activity weekly duration score + 4.0 × total moderate activity weekly duration score + 3.3 × total walking weekly duration score (40).

### Hypothetical interventions

The parametric g-formula was used to estimate cognitive impairment risks under each of the following hypothetical interventions. Single interventions were as follows:

1. Depression: Intervention resolved all depressive symptoms in all participants.
2. NSD: Intervention resulted in all participants sleeping for 6 h per night.
3. SA: Intervention ensured that all participants took part in SA.
4. IA: Intervention ensured that all participants took part in IA.

Joint interventions consisted of combinations of single interventions, which were significant in the last stage of analysis.

### Statistical analysis

The 7-year cumulative risks of cognitive impairment under hypothetical interventions were estimated by applying the parametric g-formula. The g-formula directly models probabilities for a given outcome conditional upon covariates and exposures/interventions (32). For real-world datasets, directly modeling all conditional probabilities is not feasible, especially in the presence of continuous covariates. The parametric g-formula is an extension of the g-formula, where parametric models are used to model probabilities, instead of direct calculations. Under the assumptions of no unmeasured confounding and no model misspecification, this method can provide an estimate of the risk of outcomes under full adherence to different hypothetical sustained interventions (27). The standardized risk is estimated by a weighted average of the risks of cognitive impairment conditional on the given intervention and observed confounder history. The weights are probability distribution functions of the time-varying confounders estimated using parametric regression models. The weighted average is approximated through the Monte Carlo simulation (41). We used cognitive impairment as the outcome and simulated cognitive impairment risks under each single and joint intervention using the parametric g-formula with the following four-step algorithm (42):

1. Fit a parametric regression model for each time-varying covariate, as a function of the baseline covariate and covariate history among participants followed up to time *k*.
2. Estimate the conditional probability of cognitive impairment, as a function of conditional on intervention and covariate history and surviving and remaining uncensored among participants followed up to time *k*, using a pooled over-time logistic regression model to approximate time-to-failure risk.
3. Perform a Monte Carlo simulation to generate life histories for a pseudo-population of 10,000 simulated individuals, in which baseline covariates are randomly sampled with replacements from the original population. Time-varying covariates are drawn from the parametric distribution in Step 1, and intervention covariates are set based on the defined strategy, except for the natural course (no intervention) strategy.
4. Compute the intervention risk estimate of cognitive impairment at 7 years in the pseudopopulation. The risk ratio (RR)/risk difference (RD) can be constructed between each strategy and the natural course. Ninety-five percent confidence intervals (CIs) are computed by repeating the entire aforementioned steps in 500 bootstrap samples.

The aforementioned steps were repeated for each single and joint intervention to compute cognitive impairment risks, RRs, and RDs. We also conducted subgroup analyses to assess different effects of each single intervention according to gender (male and female) and education (illiteracy and literacy). To test the model reliability, sensitivity analyses were performed by changing the order of time-varying covariates. All analyses were conducted using the R 4.1.2 statistical package for the g-formula for Windows.

## Results

### Baseline characteristics

Table 1 summarizes the sample characteristics at baseline. The mean age of respondents was 68.0 years, with an SD of 6.6 years. The proportion of men was larger than that of women: 54.9% and 45.1%, respectively. Among the population, 71.4% were literate and 81.6% were married. Over 75% were classified as being without restricted ADLs (79.4%) and IADLs (77.3%) at baseline. As regards chronic diseases, 52.0% of the individuals had 1–2 such diseases and 22.0% of them had more than two. Significantly more participants had never smoked (53.9%) or drank (66.9%) than participating in those actions. The mean CES-D-10 score and NSD were 8.5 points and 6.3 h, respectively. Accordingly, the prevalence of individuals without depression and of those who sleep 6 h at night was 37.9% and 22.6%, respectively. As regards leisure activity engagement, the mean score of PA was 103.5. The prevalence of older adults participating in SA and IA was 40.7% and 19.4%, respectively.

**Table 1:** The distribution of variables at baseline.

**Characteristic**	**N (%)**
Age (years, mean ± SD)	68.0 ± 6.6
60~	1,875 (38.9)
65~	1,229 (25.5)
70~	875 (18.2)
75~	499 (10.3)
80~	341 (7.1)
**Gender (** ***n*** **/%)**	
Male	2,647 (54.9)
Female	2,172 (45.1)
**Education level (** ***n*** **/%)**	
Illiterate	1,378 (28.6)
Literate	3,441 (71.4)
**Marital status (** ***n*** **/%)**	
Married	3,932 (81.6)
Others	887 (18.4)
**ADLs (** ***n*** **/%)**	
Restricted	994 (20.6)
Unrestricted	3,825 (79.4)
**IADLs (** ***n*** **/%)**	
Restricted	1,094 (22.7)
Unrestricted	3,725 (77.3)
**Number of chronic diseases (** ***n*** **/%)**	
None	1,252 (26.0)
1–2	2,504 (52.0)
≥3	1,063 (22.0)
**Smoking status (** ***n*** **/%)**	
None	2,596 (53.9)
Still smokes	1,614 (33.5)
Quit	609 (12.6)
**Drinking status (** ***n*** **/%)**	
None	3,225 (66.9)
Drink but less than once a month	380 (7.9)
Drink more than once a month	1,214 (25.2)
**CES-D 10 (score, mean** ±**SD)**	8.5 ± 6.1
With depression	1,827 (62.1)
Without depression	2,992 (37.9)
**NSD (hours, mean** ±**SD)**	6.3 ± 1.9
6 h	1,091 (22.6)
Others	3,728 (77.4)
**SA (** ***n*** **/%)**	
Participate	1,959 (40.7)
Not participate	2,860 (59.3)
PA (score, mean ± SD)	103.5 ± 77.6
**IA (** ***n*** **/%)**	
Participate	935 (19.4)
Not participate	3,884 (80.6)

ADLs, activities of daily living scores; IADLs, instrumental activities of daily living scores; NSD, nighttime sleep duration; SA, social activity; PA, physical activity; IA, intellectual activity.

During the 7-year follow-up, 1,808 participants developed cognitive impairment, with a significantly lower prevalence in men than in women (28.9% vs. 48.1%); similarly, this tendency was observed in the literate and illiterate groups, with 25.3% and 68.1% prevalence, respectively.

### Interventions in total population

The 7-year cumulative risks of cognitive impairment under four single hypothetical interventions, as estimated by the parametric g-formula, are shown in Table 2. The observed cognitive impairment risk was 37.52%, and the simulated risk in the natural course was 39.28% (95% CI: 35.99–44.12). Among the independent hypothetical interventions, participants who were in the IA intervention group showed the greatest risk reduction, with an RR of 0.75 (95% CI: 0.67–0.82). Notably, independent interventions for depression and NSD showed similar effects, with RRs of 0.89 (95% CI: 0.85–0.93) and 0.88 (95% CI: 0.80–0.95), respectively. However, participants who were in the SA intervention group did not show a decreased risk of cognitive impairment, with an RR of 1.00 (95% CI: 0.95–1.04).

**Table 2:** Cognitive impairment risks at 7-year follow-up under natural course and single hypothetical interventions.

**Intervention**	**Risk (%)**	**95% CI**	**RR**	**95% CI**	**RD (%)**	**95% CI**
Natural course	39.28	35.99, 44.12	1.00			
Depression	34.96	31.01, 39.79	0.89	0.85, 0.93	−4.33	−5.85, −3.08
NSD	34.50	29.73, 40.03	0.88	0.80, 0.95	−4.78	−7.75, −2.12
SA	39.12	35.02, 44.16	1.00	0.95, 1.04	−0.16	−1.99, 1.59
IA	29.35	25.38, 34.01	0.75	0.67, 0.82	−9.94	−13.29, −7.03

NSD, nighttime sleep duration; SA, social activity; IA, intellectual activity, RR, risk ratio; RD, risk difference; CI, confidence interval.

Table 3 depicts the cognitive impairment risks under different combinations of joint interventions, after exclusion of the SA intervention due to its ineffectiveness. All joint interventions could significantly reduce cognitive impairment risks among older adults. Due to the similar effects of independent interventions on depression and NSD, the combination of “Depression + IA” and “NSD + IA” showed similar effects, with RRs of 0.66 (95% CI: 0.58–0.73) and 0.65 (95% CI: 0.58–0.70), respectively. The joint intervention of “Depression + NSD” could reduce the risk by 8.93%, with an RR of 0.77 (95% CI: 0.69–0.84). The “All factors” joint intervention, that combined interventions on depression, NSD, and IA, reduced the risk by 17.11%, with an RR of 0.56 (95% CI: 0.48–0.65).

**Table 3:** Cognitive impairment risks at 7-year follow-up under natural course and joint hypothetical interventions.

**Intervention**	**Risk (%)**	**95% CI**	**RR**	**95% CI**	**RD (%)**	**95% CI**
Natural course	39.28	35.99, 44.12	1.00			
Depression + NSD	30.36	25.68, 35.75	0.77	0.69, 0.84	−8.93	−12.11, −6.27
Depression + IA	25.89	21.99, 30.77	0.66	0.58, 0.73	−13.39	−16.78, −10.56
NSD + IA	25.51	21.69, 28.69	0.65	0.58, 0.70	−13.92	−17.02, −12.02
All factors	22.17	17.81, 27.40	0.56	0.48, 0.65	−17.11	−20.75, −13.91

NSD, nighttime sleep duration; IA, intellectual activity, RR, risk ratio; RD, risk difference; CI, confidence interval.

### Interventions in subgroups

**Figure 2:**
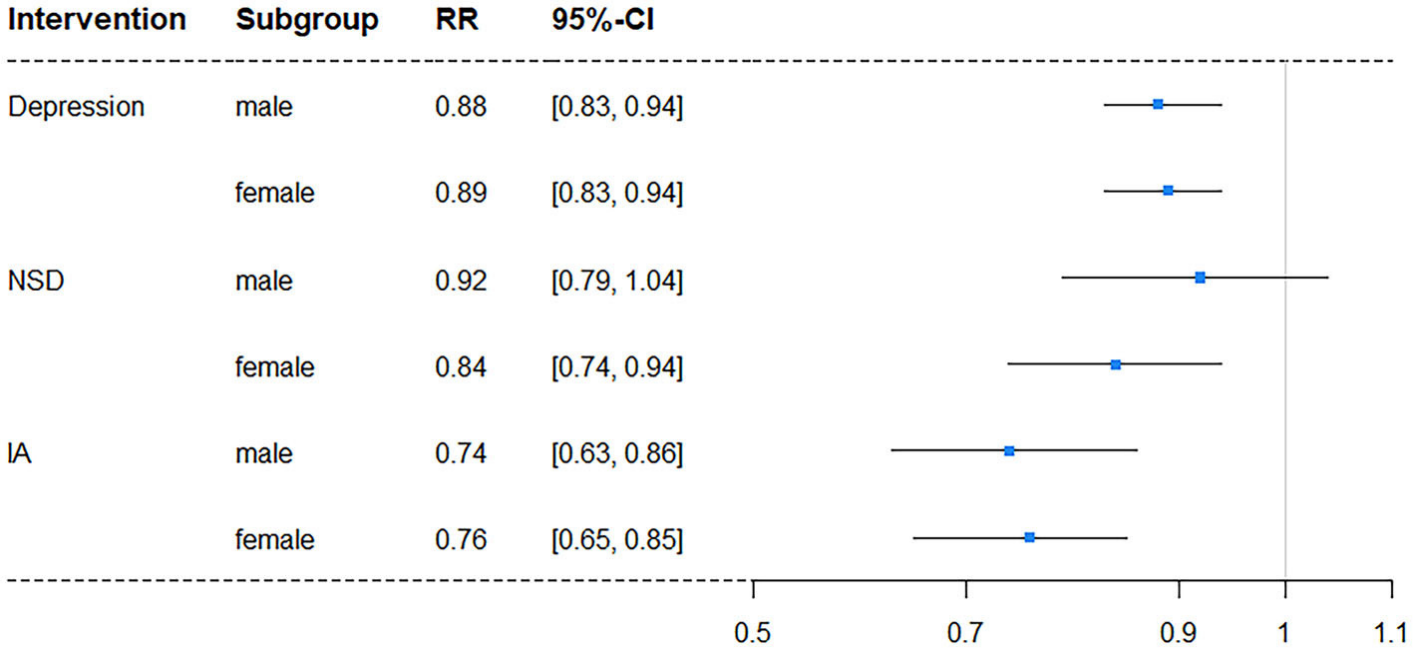
Forest plot of RR for depression risk in subgroups stratified by gender (NSD, nighttime sleep duration; IA, intellectual activity, RR, risk ratio; CI, confidence interval).

**Figure 3:**
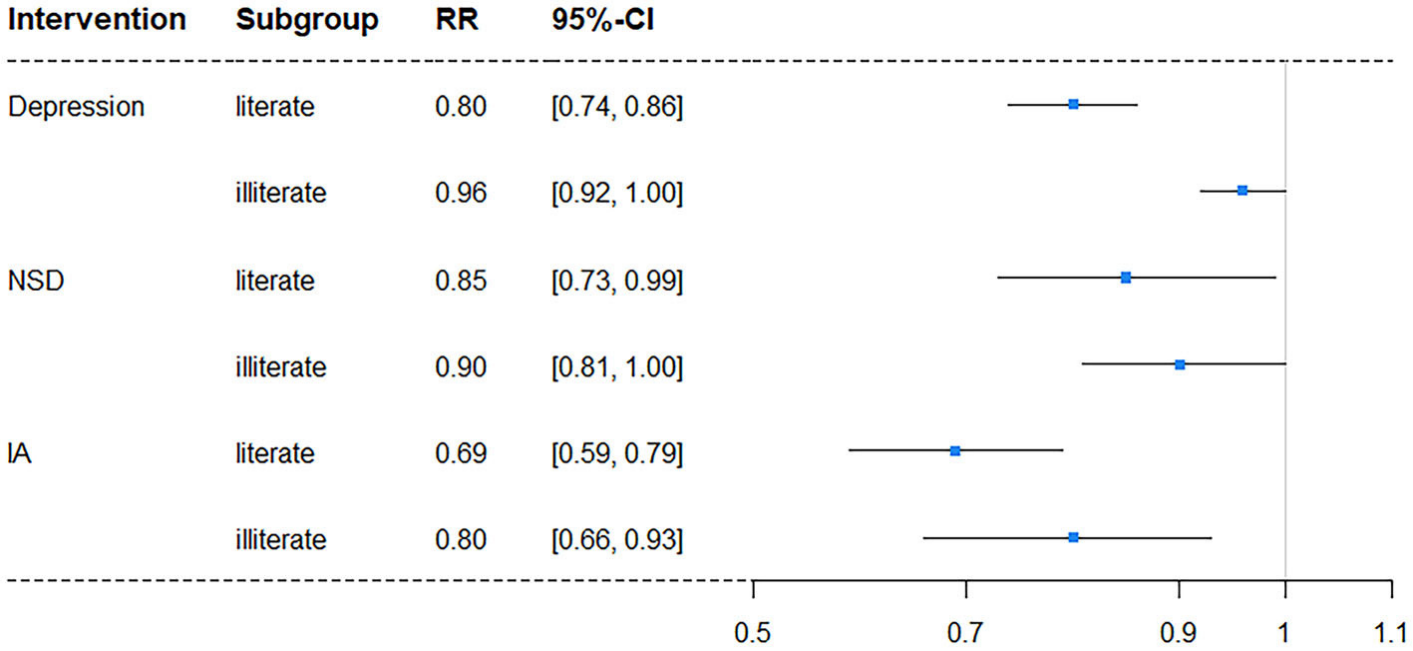
Forest plot of RR for depression risk in subgroups stratified by education level (NSD, nighttime sleep duration; IA, intellectual activity, RR, risk ratio; CI, confidence interval).

In subgroup analyses, intervention on SA was excluded once more due to its lack of significance. The results showed a difference in independent intervention when stratified by gender (Table 4) and education level (Table 5). The cognitive impairment risk in men was lower than that in women (33.83% vs. 49.48%) in the natural course, similar to the literate and illiterate groups (26.79% vs. 72.62%). For gender subgroups, in men, only independent interventions on depression and IA had significant effects on reducing the risks of cognitive impairment, with RRs of 0.88 (95% CI: 0.83–0.94) and 0.74 (95% CI: 0.63–0.86), respectively. In contrast, for women, all three independent interventions on depression, NSD, and IA had significant effects, with RRs of 0.89 (95% CI: 0.83–0.94), 0.84 (95% CI: 0.74–0.94), and 0.76 (95% CI: 0.65–0.85), respectively (Figure 2). For education subgroups, in the literate group, all three independent interventions on depression, NSD, and IA had significant effects, with RRs of 0.80 (95% CI: 0.74–0.86), 0.85 (95% CI: 0.73–0.99), and 0.69 (95% CI: 0.59–0.79), respectively. Similarly, in the illiterate group, all three independent interventions on depression, NSD, and IA had significant effects, with RRs of 0.96 (95% CI: 0.92–1.00), 0.90 (95% CI: 0.81–1.00, and 0.80 (95% CI: 0.66–0.93), respectively (Figure 3).

**Table 4:** Cognitive impairment risks at 7-year follow-up under natural course and single hypothetical interventions in subgroups stratified by gender.

**Intervention**	**Risk (95% CI)**		**RD (95% CI)**	
	**Male**	**Female**	**Male**	**Female**
Natural course	33.83 (27.24, 40.13)	49.48 (42.91, 55.58)		
Depression	29.91 (23.95, 36.02)	43.93 (36.79, 50.15)	−3.92 (−5.57, −2.03)	−5.55 (−8.03, −3.14)
NSD	31.23 (23.78, 38.62)	41.44 (33.56, 49.48)	−2.61 (−7.01, 1.17)	−8.03 (−12.57, −3.03)
IA	24.95 (19.19, 31.46)	37.54 (29.50, 45.11)	−8.89 (−12.54, −4.87)	−11.94 (−17.27, −7.28)

NSD, nighttime sleep duration; IA, intellectual activity, RD, risk difference; CI, confidence interval.

**Table 5:** Cognitive impairment risks at 7-year follow-up under natural course and single hypothetical interventions in subgroups stratified by education.

**Intervention**	**Risk (95% CI)**		**RD (95% CI)**	
	**Illiteracy**	**Literacy**	**Illiteracy**	**Literacy**
Natural course	72.62 (64.53, 79.40)	26.79 (21.65, 32.38)		
Depression	69.54 (60.57, 77.94)	21.52 (16.93, 26.69)	−3.09 (−5.71, −0.31)	−5.27 (−7.08, −3.58)
NSD	65.31 (54.82, 75.64)	22.90 (17.44, 29.57)	−7.32 (−13.32, −0.62)	−3.89 (−7.06, −3.72)
IA	58.21 (45.83, 70.26)	18.51 (13.62, 23.95)	−14.41 (−24.19, −4.96)	−8.29 (−11.07, −5.49)

NSD, nighttime sleep duration; IA, intellectual activity, RD, risk difference; CI, confidence interval.

## Discussion

We estimated the 7-year cumulative risks of cognitive impairment under single and joint interventions on depression, sleep duration, and leisure activity engagement among older Chinese community-dwelling adults by applying the parametric g-formula to the nationally representative CHARLS cohort. The results of the present study suggest that single interventions on depression, NSD, and IA could reduce cognitive impairment risks, and that different combinations of these interventions reduced the risk further. Similar effects were observed in gender subgroups, with interventions on depression and IA reducing cognitive impairment risks. However, the effects of interventions on depression and IA were greater in literate than illiterate individuals.

The results of the present study showed that independent hypothetical interventions on depression, NSD, and IA could reduce cognitive impairment risks by 4.33%, 4.79%, and 9.94%, respectively, while the joint hypothetical intervention of all three factors could significantly reduce cognitive impairment risk by 17.11%. These findings confirmed the conclusions of earlier studies, wherein depression, inappropriate NSD, and limited IA were found to be risk factors for cognitive impairment among older adults (11–13). As the g-formula is a causal-effect analytic method, the results of the present study showed the causal effects of depression, inappropriate NSD, and limited IA engagement on the incidence of cognitive impairment. Thus, the social and public health implications of this finding are that it can provide both a theoretical and data-driven basis for future RCTs of interventions on depression, inappropriate NSD, and limited IA engagement to prevent cognitive impairment among older adults. Moreover, our result is also helpful for community health service institutions to provide timely intervention for community-dwelling older adults with depression, inappropriate NSD, and limited IA engagement to reduce the risk of cognitive impairment. Nonetheless, it is unclear how these factors might increase cognitive impairment risks. Currently, researchers proposed several depression-relevant mechanisms as follows: (1) depressive symptoms can generate hyperactivity of the hypothalamic–pituitary–adrenal axis, thus increasing glucocorticoids, possibly leading to hippocampal damage and development of cognitive impairment (43). (2) Depression can cause hippocampal atrophy and accelerate the loss in hippocampal volume in women *via* the primary mediating mechanism of glucocorticoids (44). (3) Depression may also contribute to a cognitive decline through other pathways such as vascular disease, inflammation, impact on nerve growth factors, or by increasing β-amyloid accumulation (45). As regards the impact of NSD on cognition, evidence shows that the mechanisms of short and long sleep durations are different. Short sleep duration contributes to cognitive impairment *via* several different pathologies such as impaired β-amyloid clearance, pathological tau, impaired synaptic plasticity, atrophy of the cortex, and circadian rhythm disturbances. In contrast, long sleep duration is relevant to sleep fragmentation and chronic inflammation, which are linked to lower cognition (46). Moreover, Spira et al. (47) demonstrated that, among individuals with a normal cognitive function, sleep durations of < 7 h and of >7 h may accelerate frontotemporal gray matter atrophy and subsequently increase the cognitive impairment risk. This result is not consistent with the results from both this study and an earlier one, which took 6 h as the reference sleep duration (12). These differences may have arisen due to differences in the study population such as race, study sample size, and population age bracket. The beneficial effects of IA engagement on impaired cognition risk reduction may be due to improved cognitive reserves. Excess β-amyloid can accumulate in the brain as plaques that block the gaps between synapses, which in turn affects the electrical signals used by the neurons to communicate with each other; moreover, the chemistry-imbalanced tau protein can entangle with other tau proteins to form tangles, which will destroy microtubules and prevent necessary nutrients from reaching nerve endings of neurons, thus causing the entire cell to become dysfunctional. All of these alterations can cause cognitive impairment (48). According to the cognitive reserve theory, IA can strengthen the functioning and plasticity of neural circuits, further increasing the cognitive reserve and decreasing the risk of cognitive impairment (25).

Cognitive impairment risks did not decrease under the SA intervention. A similar result was reported by Li et al. (48) who found that a higher participation in SA did not improve cognitive function. Moreover, an RCT conducted by Park et al. (49) also found that engagement in SA had limited cognitive benefits. Interpretations for this phenomenon may be attributed to the fact that reduced social participation is an early manifestation of cognitive impairment rather than a cause (48). Additionally, simple SA is not particularly helpful in optimizing cognition in the long term because of the relatively passive participation and low cognitive demand. Such passivity may not be particularly beneficial for enhancing cognitive reserve (50). Therefore, monitoring changes of engagement in SA, rather than intervening to increase SA participation, may be beneficial to prevent cognitive impairment among older adults *via* timely identification and intervention.

Notably, in subgroup analyses, although similar effects of interventions on depression and IA for cognitive impairment risks were observed in gender subgroups, interventions on depression and IA displayed stronger effects in literate than illiterate participants. Several studies reported that education years and high education levels could predict and alleviate the severity of depression (51, 52). Accordingly, the intervention on depression was effective for literate individuals, reducing cognitive impairment risks. As regards different effectiveness levels of interventions on IA for literate and illiterate individuals, as far as we know, we are the first to find a difference. We speculate that older adults with high education levels get more training and application during engagement because IA subtypes need more comprehend ability. As a result, intervention on IA was more effective for literate than for illiterate individuals. Recently, studies investigated the effectiveness of continuing education for the prevention of cognitive impairment and dementia. Thow et al. (53) reported that older adults who attended university courses over a period of 12 months showed significant improvement in their language processing capacity compared with healthy adult controls, but there was no change in their fluid cognitive function. However, another study, by Lenehan et al. (54), found that 92.5% of older individuals who undertook further education in 12-month part- or full-time university courses displayed a significantly linear increase in cognitive reserve over the 4 years of study, indicating that continuing education could improve cognitive function and offset cognitive decline. Because of these contradicting results, more studies focusing on the effectiveness of continuing education for the prevention of cognitive impairment and dementia are essential. If the possible preventive effects are confirmed, then interventions based on continuing education for older adults should be established and promoted in communities and nursing facilities.

The strengths of the present study are the population-based longitudinal design, standardized survey methods, rigorous case validation, and long follow-up time. We used the parametric g-formula to appropriately adjust for time-varying confounders and estimate the effects of independent and joint interventions on depression, NSD, and IA for a reduction in cognitive impairment risks. Thus, the estimates are more directly relevant and provide more information to guide public health and clinical decisions. However, the present study has several limitations. First, the validity of the g-formula approach relies on three common assumptions for observational research: no model misspecification, no measurement error, and no unmeasured confounding. We adjusted for many potential risk factors to alleviate the “no measurement error” and “no unmeasured confound issues,” which were inevitable for this observational study. Evidence that simulated data under no intervention using the parametric g-formula were analogous to the observed data, indicating the condition of no model misspecification. Our sensitivity analyses also revealed that our results were robust across different specifications. Second, the precondition of generalizing our results to other populations is based on the condition that the target population should have the same distribution of effect modifiers and interference patterns as our study population because the calculation principle of the g-formula is based on the standardized risk to the distribution of risk factors. Due to the advantage of a population-based sample, we consider that the conclusion of this study has a certain degree of generalizability to some extent. Finally, we implicitly assumed that the counterfactual outcome for all scenarios should be the same as the observed outcome under the observed exposure history. It should be noted that, in real life, idealized intervention effects and complete adherence of participants are hard to accomplish; therefore, our estimates purely correspond to the best-case scenario, rather than the specific circumstances of each program.

## Conclusions

By applying the parametric g-formula to a longitudinal sample from CHARLS, we found that hypothetical interventions on depression, NSD, and IA can reduce cognitive impairment risks among older Chinese adults, both independently and jointly. Furthermore, we observed similar effects of interventions on depression and IA in gender subgroups, but interventions on depression and IA had stronger effects in literate than illiterate individuals. Thus, the findings of the present study suggest that intervention measures for depression, inappropriate NSD, and limited intellectual activities and any combination thereof may prove to be effective strategies for the prevention of cognitive impairment among older adults.

## Data availability statement

The original contributions presented in the study are included in the article/supplementary material, further inquiries can be directed to the corresponding author.

## Ethics statement

The studies involving human participants were reviewed and approved by the Biomedical Ethics Committee, Peking University. Written informed consent to participate in this study was provided by the participants' legal guardian/next of kin.

## Author contributions

NZ: conceptualization, data curation, statistical analysis, methodology, drafting, manuscript editing, and graphics production. FC and CW: methodology and reviewing. PY: conceptualization, methodology, supervision, reviewing, and editing. All authors contributed to the article and approved the submitted version.
